# Efficacy and Safety of Nitazoxanide, Albendazole, and Nitazoxanide-Albendazole against *Trichuris trichiura* Infection: A Randomized Controlled Trial

**DOI:** 10.1371/journal.pntd.0001685

**Published:** 2012-06-05

**Authors:** Benjamin Speich, Shaali M. Ame, Said M. Ali, Rainer Alles, Jan Hattendorf, Jürg Utzinger, Marco Albonico, Jennifer Keiser

**Affiliations:** 1 Department of Medical Parasitology and Infection Biology, Swiss Tropical and Public Health Institute, Basel, Switzerland; 2 University of Basel, Basel, Switzerland; 3 Public Health Laboratory (Pemba) - Ivo de Carneri, Chake Chake, Tanzania; 4 Division of Pharmaceutical Technology, Department of Pharmaceutical Sciences, University of Basel, Basel, Switzerland; 5 Department of Epidemiology and Public Health, Swiss Tropical and Public Health Institute, Basel, Switzerland; 6 Ivo de Carneri Foundation, Milano, Italy; Yale Child Health Research Center, United States of America

## Abstract

**Background:**

The currently used anthelmintic drugs, in single oral application, have low efficacy against *Trichuris trichiura* infection, and hence novel anthelmintic drugs are needed. Nitazoxanide has been suggested as potential drug candidate.

**Methodology:**

The efficacy and safety of a single oral dose of nitazoxanide (1,000 mg), or albendazole (400 mg), and a nitazoxanide-albendazole combination (1,000 mg–400 mg), with each drug administered separately on two consecutive days, were assessed in a double-blind, randomized, placebo-controlled trial in two schools on Pemba, Tanzania. Cure and egg reduction rates were calculated by per-protocol analysis and by available case analysis. Adverse events were assessed and graded before treatment and four times after treatment.

**Principal Findings:**

Complete data for the per-protocol analysis were available from 533 *T. trichiura*-positive children. Cure rates against *T. trichiura* were low regardless of the treatment (nitazoxanide-albendazole, 16.0%; albendazole, 14.5%; and nitazoxanide, 6.6%). Egg reduction rates were 54.9% for the nitazoxanide-albendazole combination, 45.6% for single albendazole, and 13.4% for single nitazoxanide. Similar cure and egg reduction rates were calculated using the available case analysis. Children receiving nitazoxanide had significantly more adverse events compared to placebo recipients. Most of the adverse events were mild and had resolved within 24 hours posttreatment.

**Conclusions/Significance:**

Nitazoxanide shows no effect on *T. trichiura* infection. The low efficacy of albendazole against *T. trichiura* in the current setting characterized by high anthelmintic drug pressure is confirmed. There is a pressing need to develop new anthelmintics against trichuriasis.

**Trial Registration:**

Controlled-Trials.com ISRCTN08336605

## Author Summary

More than 5 billion people are at risk of infection with one of the three most common intestinal worms, the roundworm *Ascaris lumbricoides*, the whipworm *Trichuris trichiura*, and two different kinds of hookworms. The global strategy to control these intestinal worm infections is through the regular administration of deworming drugs to school-aged children (albendazole, 400 mg; mebendazole, 500 mg). However, especially against *T. trichiura*, a low treatment response is observed with single doses of both drugs. We tested the antiprotozoal drug nitazoxanide, which had shown promising trichuricidal properties in *in vitro* experiments. A randomized controlled trial was carried out on the island of Pemba in Tanzania. Four treatment arms were included: (i) single albendazole (400 mg), (ii) single nitazoxanide (1,000 mg), (iii) nitazoxanide-albendazole combination (1,000 mg–400 mg) with each drug given separately on two consecutive days, and (iv) placebo. Children were asked for adverse events at several time points after treatment. Nitazoxanide showed no ability to cure *T. trichiura-*infected children and caused significantly more mild adverse events than placebo. Albendazole and the nitazoxanide-albendazole combination showed only a minimal effect against *T. trichiura*. Our results emphasize the urgent need to develop new, safe, and effective anthelmintic drugs against *T. trichiura*.

## Introduction

Infections with the soil-transmitted helminths, *Ascaris lumbricoides*, *Trichuris trichiura*, and the two hookworm species *Ancylostoma duodenale* and *Necator americanus*, are the most common infections of humans, causing an estimated global burden of 39 million disability-adjusted life years lost (DALYs) [1]–[3]. Globally more than 5 billion people are at risk and at least 1 billion people are currently infected with one or several of these nematodes [1], [3], [4]. On Pemba, soil-transmitted helminth infections remain of considerable public health importance with particularly high prevalences observed for *T. trichiura* and hookworm [5].

Preventive chemotherapy targeting at-risk communities (i.e., school-aged children) is in place in many countries. These programs aim at morbidity control, and hence intensity of infection is kept below a threshold of disease [6]. The two benzimidazoles, albendazole and mebendazole, as well as pyrantel pamoate and levamisole are recommended drugs against infections with soil-transmitted helminths [7], [8]. These drugs have been widely used as they were developed and put on the market between 1966 (pyrantel pamoate) and 1980 (albendazole) [9]. The four drugs exhibit distinct differences in their therapeutic profile, with the exception of considerably high efficacy of all drugs against *A. lumbricoides*. For the treatment of hookworm infection, only albendazole achieves satisfactory cure rates when administered as a single oral dose. Of particular concern is the situation for *T. trichiura*; single doses of mebendazole show the highest cure rates, but these are usually low or only moderate (<50%) [10]. The pressing need for developing new anthelmintic drugs, particularly for *T. trichiura*, cannot be emphasized enough. In addition, the therapeutically useful life span of these drugs is endangered should resistance develop and start to spread [11]–[13].

The antiprotozoal drug nitazoxanide, which is marketed for the treatment of intestinal parasitic infections (i.e., *Cryptosporidium parvum* and *Giardia intestinalis*) has been reported to also have trichuricidal properties [14], [15]. Silbereisen and colleagues recently showed that nitazoxanide is highly active against *Trichuris muris in vitro* [16]. In clinical trials high cure rates were reported against *T. trichiura* as well as *A. lumbricoides* and hookworm, following multiple doses (200 mg for children aged below 12 years and 500 mg for patients aged 12 years and above, twice daily for 3 days) of nitazoxanide [17]–[19]. Hence, nitazoxanide has been listed as a potential drug candidate for human-soil transmitted helminthiasis and further research was suggested [20].

**Figure 1:**
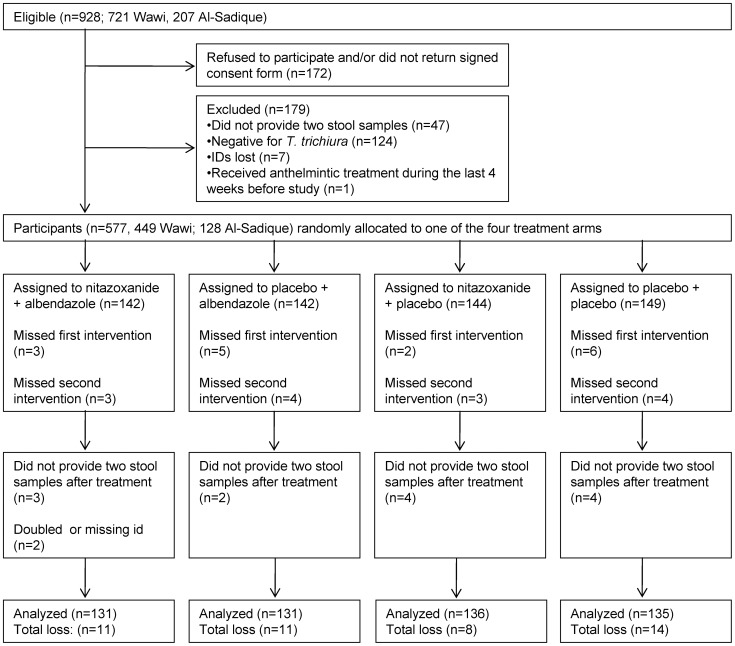
Flow diagram illustrating the compliance in the randomized controlled trial. Flow diagram of the randomized, placebo-controlled trial assessing the efficacy of albendazole, nitazoxanide, and albendazole combined with nitazoxanide, administered separately on two consecutive days, in the treatment of *Trichuris trichiura* infections.

We report the findings of a randomized, double-blind, placebo-controlled trial, which specifically assessed the efficacy and safety of nitazoxanide given as a single dose (1,000 mg) in the treatment of *T. trichiura* in school-aged children on Pemba, Tanzania. The standard treatment with albendazole (400 mg) served as a benchmark. In addition, one group of children was given nitazoxanide (1,000 mg) on the first day and albendazole (400 mg) on the following day, in order to evaluate if an enhanced efficacy would be observed following this combination chemotherapy. A fourth group of children received placebo (the treatment groups are summarized in Figure 1). Since *A. lumbricoides* and hookworm coexist in the current setting, outcomes on these nematodes are also reported.

## Methods

### Ethics Statement

Ethical clearance was obtained from the ethics committee of Basel, Switzerland (EKBB, reference no. 225/10) and from the Ministry of Health and Social Welfare in Zanzibar (ZAMREC, reference no. 0001/010). The study is registered at Current Controlled Trials (ISRCTN08336605). Written informed consent was acquired from the children's parents or legal guardians to participate in this trial. Children assented orally. It was emphasized that study participation would be voluntary and withdrawal possible at any time.

### Study Setting

Our trial was carried out in June and July 2011 on Pemba, one of the major islands in the Zanzibar archipelago, which belongs to Tanzania. The schools of Wawi (geographical coordinates; 5°15′22″S latitude, 39°47′28″E longitude) and Al-Sadik (5°15′42″S, 39°48′25″E), both located less than 10 km from Chake Chake, the main town of Pemba (5°14′45″S, 39°45′00″E, ∼22,000 inhabitants), were selected. In the school year 2010/2011, a total of 1,100 children were registered in Wawi and 437 in Al-Sadik. Both schools were easily accessible by car from the Public Health Laboratory–Ivo de Carneri (PHL–IdC) in Chake Chake.

### Sample Size Calculation

To determine the sample size, we assumed a cure rate of 28% for single oral albendazole against *T. trichiura* based on data from a meta-analysis [21]. The efficacy of single oral nitazoxanide against *T. trichiura* is not known. Therefore we assumed that it would be at least similar to that of albendazole (20–30%). Moreover, we hypothesized that an albendazole-nitazoxanide combination would achieve a considerably higher cure rate (50%). Monte Carlo simulations (imperfect test with a sensitivity of 90%) computed a sample size of 95 *T. trichiura*-infected individuals in each arm to detect a difference of single medication treatments *versus* both, the placebo group and the albendazole-nitazoxanide group at a significance level of 5% with 80% power. Allowing for drop outs and assuming an overall *T. trichiura* prevalence of 80%, we targeted approximately 125 children per treatment arm for the baseline screening.

### Study Flow and Procedures

Before the onset of the study, the headmasters of Wawi and Al-Sadik were asked for permission to carry out the trial at their schools. The parents of the children were invited to the schools, so that they could be explained the purpose and procedures of the study, including potential risks and benefits. Questions could be asked in a discussion round and clarification was given to parents.

At the first day of enrolment, all children attending standards one to five received an empty stool container together with a consent form; both labeled with unique identification numbers (ID). After recording their name, sex, age, and school grade, children were invited to return the container with a fresh sample of the next morning stool together with the signed consent form. Filled stool containers and signed consent forms were collected from the children and a new empty container was handed out for collection of a second stool sample on the following day.

All children who returned a signed informed consent and had two stool samples were assigned to one of the four treatment groups, regardless of their helminths infection status. Before drug administration, all children were examined by a physician. Exclusion criteria were: (i) presence of any abnormal medical condition, judged by the study physician; (ii) history of acute or severe chronic disease (cancer, diabetes, chronic heart, liver, or renal disease); and (iii) recent use of anthelmintic drugs (within past 4 weeks). Additionally, the weight and height of all children were measured and if there was any indication of fever, axillary temperature was taken using a digital thermometer.

Three weeks posttreatment, children were asked again for two stool samples collected over consecutive days to determine the efficacy of the different treatments. At the end of the study, all children who were still infected with soil-transmitted helminths were offered a dose of albendazole (400 mg) following national guidelines [7], [8].

### Randomization, Treatment, and Adverse Events

An independent statistician created a randomization code assigning to each ID a number from 1–4, representing the four treatment arms: (i) nitazoxanide (1,000 mg) plus albendazole (400 mg); (ii) nitazoxanide-matching placebo plus albendazole (400 mg); (iii) nitazoxanide (1,000 mg) plus albendazole-matching placebo; and (iv) two placebos. For blinding purposes, the tablets were packed before treatment into small plastic bags, labeled with the unique IDs. Nitazoxanide (Alinia®) and albendazole (Zentel®) tablets were the products of Romark and GlaxoSmithKline, respectively. Placebos were produced at the Department of Pharmaceutical Sciences, University of Basel by one of the authors (R.A.). During the trial all drugs were stored at room temperature, not exceeding 25°C. Since interactions between nitazoxanide and albendazole have not been studied before, the two drugs were administered on two consecutive days. Hence, on the first day of treatment, children received two tablets of nitazoxanide (500 mg each) or two nitazoxanide-matching placebos. On the second day, children received one tablet of albendazole (400 mg) or an albendazole-matching placebo. Since the trial was double-blinded, neither the child, nor the person giving the treatment knew to which treatment arm the child was allocated to.

Before treatment, children were asked if they suffer from any adverse events. Drugs were then administered with a cup of water and each child received a small snack. Three hours after treatment, adverse events were actively assessed by interviewing each child. On the following day, before receiving the second treatment, adverse events were investigated again. The same procedures were repeated 3 and 24 hours after the second treatment.

### Parasitological Analysis

Stool samples were transferred to PHL–IdC. Duplicate Kato-Katz thick smears, using 41.7 mg templates, were prepared from each stool sample [22]. Kato-Katz thick smears were examined under a microscope by experienced laboratory technicians within 20–40 min after preparation, as recommended to avoid over clearing of hookworm eggs [23]. All hookworm eggs were counted. Subsequently, the slides were re-examined for *T. trichiura* and *A. lumbricoides*, with parasite eggs counted and recorded separately. To ensure high quality of the diagnosis, 10% of the slides, selected at random, were re-examined. In cases of discordant results, slides were read a third time and results discussed until agreement was reached.

### Statistical Analysis

Parasitological data and reported adverse events were double entered into an Excel spreadsheet and cross-checked. In case of discrepancy, the original files were consulted to correct the data entry. All statistical analyses were performed with Stata 10.1 software (StataCorp).

Cure rates were calculated as the proportion of egg-positive children at baseline, who became egg-negative after treatment. Egg counts from the four Kato-Katz thick smears were added up for each species and multiplied by a factor 6 and expressed as eggs per gram of stool (EPG). Infection intensity was classified using pre-defined cut-offs by the World Health Organization (WHO; *T. trichiura* light, 1–999 EPG; moderate, 1,000–9,999; and heavy, ≥10,000) [24]. Differences among treatment arms concerning cure rates and observed adverse events were analyzed with logistic regressions.

Geometric mean egg counts were calculated for the different treatment arms before and after drug intervention to calculate the respective egg reduction rates (ERR). We also calculated the average dose (mg/kg) of each drug that the children received per treatment arm and analyzed with a logistic regression if the dose of treatment had an influence on the cure rates.

We used two different types of analyses: (i) per-protocol analysis, including only those children who had complete data records (quadruplicate Kato-Katz results before and after treatment and being treated); and (ii) available case analysis (which is sometimes erroneously referred to as an intention-to-treat analysis [25]) based on the treatment intent, hence analyzing data from all individuals who were assigned to one of the four treatment arms and had primary outcome data. A greater emphasis is given in our manuscript on the per-protocol analysis (available case results summarized in Table S1) since a bias might have been introduced in the available case analysis given that children absent on the first treatment day (nitazoxanide) were shifted to a later starting treatment period, while this was not possible for children missing the second treatment day (albendazole).

## Results

### Adherence

From the 928 children invited to participate in the study, 172 refused to participate or did not return a signed consent form. Another 47 children failed to provide two stool samples and from seven children the IDs on the stool samples were lost and hence they had to be excluded from the trial. One child received anthelmintic treatment less than 4 weeks before the onset of our trial and was therefore excluded. The remaining 701 children (549 from Wawi and 152 from Al-Sadik) were randomly assigned to one of the four treatment arms independently of their parasitological status. Of these, 124 were *T. trichiura*-negative and therefore excluded from the final analysis (Figure 1). Fifteen children missed the first treatment, and 14 children were absent on the second day of treatment. At follow-up, 13 children provided no or only a single stool sample and two children were excluded due to other reasons. In total 44 *T. trichiura*-infected children were lost during treatment and follow-up. Hence, 533 children were included in the per-protocol analysis. The loss of participants was distributed equally over the different treatment arms; the double placebo group was characterized by loss of the most participants (n = 14). Of the 29 participants not receiving treatment, all 14 who missed the second treatment provided primary end point data and could therefore be followed up; hence 547 children were included in the available case analysis.

### Baseline Characteristics

Most of the 701 children subjected to multiple Kato-Katz thick smears readings were diagnosed positive for *T. trichiura* (n = 577, 82%). Infections were mainly of light intensity (94%) and only one heavy *T. trichiura* infection was identified. Prevalence of hookworm and *A. lumbricoides* were 7% and 5%, respectively, and most of these infections were of light intensity. An infection with all three helminth species was diagnosed in 11 children (1.5%). The mean age of the 701 children was 10 years (range 7–15 years). Mean age, weight, and height were comparable in all four treatment arms (Table 1). There was a similar number of boys (n = 348) and girls (n = 353) participating in the study.

**Table 1:** Baseline characteristics of school-aged children included in the trial.

Characteristic	Overall	Nitazoxanide+albendazole	Only albendazole	Only nitazoxanide	Only placebo
N	701	165	177	180	179
Age mean (±SD), years	9.7 (1.6)	9.7 (1.6)	9.8 (1.7)	9.4 (1.6)	9.9 (1.6)
No. of boys/girls	348/353	82/83	85/92	94/86	87/92
No. of participants Wawi/Al Sadique	549/152	127/38	142/35	139/41	141/38
Weight mean (±SD), kg^a^	26 (5.6)	26 (5.6)	26 (5.9)	26 (5.3)	26 (5.4)
Height mean (±SD), cm^b^	130 (9.2)	130 (9.4)	130 (9.3)	129 (9.3)	130 (8.8)
**Infected with ** ***T. trichiura***					
No. infected children (%)	577 (82.3)	142 (86.1)	142 (80.2)	144 (80.0)	149 (83.2)
Geometric mean, EPG	153	147	162	148	156
Infection intensity, no (%) of infected children					
Light (1–999 EPG)	541 (93.7)	133 (93.0)	131 (92.3)	137 (95.1)	140 (94.6)
Moderate (1,000–9,999 EPG)	35 (6.1)	10 (7.0)	10 (7.0)	7 (4.9)	8 (5.4)
Heavy (≥10,000 EPG)	1 (0.2)		1 (0.7)		
**Infected with hookworm**					
No. infected children (%)	48 (6.8)	15 (9.0)	11 (6.2)	13 (7.2)	9 (5.0)
Geometric mean EPG*	36	35	37	39	35
**Infected with ** ***A. lumbricoides***					
No. infected children (%)	31 (4.4)	6 (3.6)	9 (5.1)	8 (4.4)	8 (4.5)
Geometric mean EPG	507	686	207	435	1,297
Infection intensity, no (%) of infected participants					
Light (1–4,999 EPG)	29 (93.6)	5 (83.3)	9 (100)	8 (100)	7 (87.5)
Moderate (5,000–49,000 EPG)	2 (6.6)	1 (6.7)	0	0	1 (12.5)

a 684 datasets.

b 683 datasets.

*: all infections with hookworms were classified as light.

### Efficacy against *T. trichiura*

Only very low cure rates were observed regardless of whether the sequentially administered nitazoxanide-albendazole combination, albendazole, or nitazoxanide were administered. In more detail, using the per-protocol analysis, nitazoxanide combined with albendazole achieved a cure rate of 16.0% (95% confidence interval (CI), 9.7–22.4%), whereas single doses of albendazole or nitazoxanide resulted in cure rates of 14.5% (95% CI, 8.4–20.6%), and 6.6% (95% CI, 2.4–10.8%), respectively. Children receiving placebo showed an apparent cure rate of 8.9% (95% CI, 4.0–13.8%) (Table 2). Similar results were observed using the available case analysis (Table S1). Comparing the treatment outcomes using a logistic regression revealed that albendazole had a significant effect on infections with *T. trichiura* (odds ratio (OR), 0.47; 95% CI, 0.27–0.81; p = 0.007) while nitazoxanide showed no effect (OR, 1.04; 95% CI, 0.61–1.78; p = 0.89). There was some indication of an interaction between the two drugs (OR, 0.64; 95% CI, 0.21–1.98), however, this result was not significant (p = 0.44).

**Table 2:** Effect of albendazole, nitazoxanide, sequentially administered albendazole-nitazoxanide combination, and placebo against soil-transmitted helminths.

Characteristic	Nitazoxanide+albendazole	Only albendazole	Only nitazoxanide	Only placebo
***Trichuris trichiura***				
No. of infected children	131	131	136	135
No. of children not cured after treatment	110	112	127	123
Cure rate % (95% CI)	16.0	14.5	6.6	8.9
	(9.7–22.4)	(8.4–20.6)	(2.4–10.8)	(4.0–13.8)
Geometric mean, EPG				
Before treatment	152	164	145	154
After treatment	69	89	125	127
Egg reduction rate % (95% CI*)	54.9	45.6	13.4	17.6
	(37.7–67.9)	(25.9–61.0)	(0.0–33.7)	(0.0–36.7)
**Hookworm**				
No. of infected children	14	11	12	9
No. of children not cured after treatment	2	2	4	4
Cure rate % (95% CI)	85.7	81.8	66.7	55.6
	(64.7–100.0)	(54.6–100.0)	(35.4–98.0)	(15.0–96.1)
***Ascaris lumbricoides***				
No. of infected children	5	9	8	7
No. of children not cured after treatment	0	0	3	7
Cure rate % (95% CI)	100.0	100.0	62.5	0.0
			(19.2–100.0)	
**Range of actual treatment dose (mg/kg)**				
Nitazoxanide mean (min, max)	38.2	-	38.9	-
	(22.7–64.9)		(23.9–54.1)	
Albendazole mean (min, max)	15.3	15.0	-	-
	(9.1–26.0)	(7.7–27.2)		

Effect of different treatments calculated with the per-protocol analysis.

*: 95% CI of egg reduction rates were calculated using bootstrap resampling.

The ERR for *T. trichiura* was 54.9% (bootstrap 95% CI, 37.7–67.9%) for the nitazoxanide-albendazole combination, 45.6% (95% CI, 25.9–61.0%) for albendazole alone, 13.4% (95% CI, 0.0–33.7%) for nitazoxanide alone, and 17.6% (95% CI, 0.0–36.7%) for the placebo-controlled treatment arm (Table 2). Large confidence intervals indicate a high variance in ERRs. Therefore the only significant difference between the treatment arms (defined by non-overlapping bootstrap CI), was found between nitazoxanide-albendazole compared to the nitazoxanide monotherapy treatment group and the placebo-controlled treatment arm. However, even though CIs are overlapping, there seems to be a trend that albendazole alone resulted in higher ERRs.

The amount of milligrams of drug administered per kilogram of body weight ranged from 22.7 to 64.9 mg/kg for nitazoxanide and from 7.7 to 27.2 mg/kg for albendazole (Table 2). Logistic regression revealed no statistically significant association between body weight and cure rates between the different treatment arms which received an active drug.

### Efficacy against Other Soil-Transmitted Helminths

Less than 10% of the children were infected with hookworm (n = 48; 7%) and *A. lumbricoides* (n = 31; 4%). Albendazole was highly efficacious against both parasites with cure rates of 100% against *A. lumbricoides* and 81.8% against hookworm (95% CI, 54.6–100%). Nitazoxanide showed moderate efficacy against those two nematode species with cure rates of 66.7% (95% CI, 35.4–98.0%) against hookworm and 62.5% (95% CI 19.2–100.0%) against *A. lumbricoides* (Table 2). These data, however, lacked statistical significance. Of note, placebo had an apparent cure rate against hookworm infection of 55.6%.

### Adverse Events

In total, 678 of the treated children answered a standardized questionnaire pertaining to adverse events. However, not all of these children responded at each of the five follow-up time points (Table 3). Before treatment, 28 (4.1%) children reported minor symptoms (e.g., headache and abdominal pain). After treatment (both first and second round of treatment) a total of 244 children complained at least once about minor adverse events at one of the four follow-up examinations (3 and 24 hours after each treatment). Only one child had moderate adverse events, namely headache 24 hours after receiving nitazoxanide. This child was treated with paracetamol and the headache resolved within 3 hours. In total 307 adverse events were reported after starting the treatment regimen. Abdominal cramps and headache were the most frequent ones (165 times (53.7%) and 69 times (22.5%), respectively) (Table S2). Other reported adverse events were nausea (6.8%), vertigo (5.5%), diarrhea (4.6%), fever (3.6%), allergic reaction (1.6%), vomiting (1.3%), and fatigue (0.3%).

**Table 3:** Assessed adverse events among the four treatment arms during a randomized, placebo-controlled trial carried out on Pemba Island, Tanzania.

Time point	Adverse events: none/mild				
	Percentage of people with adverse events				
	Overall	Nitazoxanide+albendazole	Albendazole	Nitazoxanide	Placebo
Before treatment	648/28	155/9	163/8	166/6	164/5
	4.1%	5.5%	4.7%	3.5%	3.0%
3 hours after first treatment	577/101	133/31*	155/16	139/34*	150/20
	14.9%	18.9%	9.4%	19.7%	11.8%
24 hours after first treatment	622/56^a^	145/19^a^	160/11	153/20	164/6
	8.3%	11.6%	6.4%	11.6%	3.5%
3 hours after second treatment	606/51	144/15	157/12	151/16	154/8
	7.8%	9.4%	7.1%	9.6%	4.9%
24 hours after second treatment	576/51	138/12	147/12	149/12	142/15
	8.1%	8.0%	7.5%	7.5%	9.6%

Adverse events were assessed at five different time points (before treatment, 3 and 24 hours after first treatment, and 3 and 24 hours after second treatment). Nitazoxanide was given on the first day of treatment, while albendazole was given on the second day of treatment.

*: statistically significant more adverse events in nitazoxanide-treated children compared to children receiving placebo.

a One of these adverse events was classified as moderate.

Three hours after the first treatment, minor adverse events were reported by 101 (14.9%) participants. Children who received placebo reported significantly more about minor adverse events compared to the pretreatment situation (OR, 2.97; 95% CI, 1.50–6.21) (Table 3). Children who were given single nitazoxanide had significantly more adverse events 3 hours after treatment compared to the placebo recipients at the same time point (OR, 2.02; 95% CI, 1.28–3.24). On the next day (24 hours after the first treatment) 56 (8.3%) children reported adverse events. Participants receiving nitazoxanide still had significantly higher odds of reporting adverse events (OR, 2.49; 95% CI, 1.38–4.50). Three hours after the second treatment, children treated with albendazole did not report significantly more adverse events than placebo recipients (OR, 1.47; 95% CI, 0.59–3.70). At this time point, nitazoxanide was no longer associated with significantly more adverse events than placebo recipients (OR, 2.04; 95% CI, 0.85–4.91) and both drugs combined (treatment group 1) showed no cumulative effect regarding adverse events (OR, 0.67; 95% CI, 0.21–2.18). Twenty-four hours after the second treatment adverse events were resolved. Logistic regression revealed ORs of 0.77 (95% CI 0.35–1.71) for albendazole, 0.76 (95% CI 0.35–1.69) for nitazoxanide, and 1.40 (95% CI 0.44–4.41) for the sequentially administered nitazoxanide-albendazole combination.

## Discussion

Following up on the promising trichuricidal properties observed in *in vitro* studies [16], we carried out the first randomized, double-blind, placebo-controlled trial administering nitazoxanide as a single dose of 1,000 mg to *T. trichiura*-infected school-aged children in a highly endemic area in Pemba. In addition, since combination chemotherapy is being advocated in many therapeutic areas, as it enhances efficacy and lowers the risk of resistance development [26], one group of children was treated with a nitazoxanide-albendazole combination which was administered over two consecutive days.

A high single dose of nitazoxanide (1,000 mg) showed no therapeutic effect against *T. trichiura*. This result is in contrast to previous studies, which reported high cure rates when the drug was administered as multiple dose treatment regimen (6 times 200 mg or 500 mg) to *T. trichiura*-infected patients [17]–[19]. It is plausible that pharmacokinetic properties of “multiple doses a day” nitazoxanide are superior to “once a day” nitazoxanide in that it achieves a longer half life. However, since the global strategy targeting neglected tropical diseases advocates preventive chemotherapy (i.e., regular deworming with single oral doses), multiple dosing is currently not recommended, as it poses operational and financial challenges [27]. A rigorous diagnostic approach as performed in this study (two times duplicate Kato-Katz thick smears before and after treatment) can lead to lower observed cure rates, since, especially light infections, are more likely to be detected [28]. Nevertheless, two of the above mentioned studies which found high cure rates and ERRs for nitazoxanide against *T. trichiura* infections also collected several stool samples after treatment, and hence pursued a thorough diagnostic approach. Diagnosis, therefore, does not seem to be the main reason for the contradictory results.

The standard treatment albendazole revealed a very low cure rate against *T. trichiura* when given at a single dose of 400 mg, which is in agreement with the results of several previous studies [21], [29], [30]. It is interesting to note that the ERR following albendazole treatment was still moderate (73%) in the same setting 12 years ago, but was similarly low as the ERR obtained in this trial, in a study conducted by Knopp et al. in 2009 in neighboring Unguja, Zanzibar [29], [30]. This might be an indicator of tolerance or resistance development to albendazole against *T. trichiura*. Of note, Levecke et al. [31] recently showed that ERRs can differ strongly between individual settings and that albendazole has higher ERRs in settings with low infection intensities. Our trial could not confirm this hypothesis since albendazole achieved only a low ERR in children characterized by low infection intensities.

The combination of albendazole and nitazoxanide had slightly, though not significantly higher cure rates and ERRs than albendazole alone. Since drug interactions have not been evaluated for this combination, drugs were administered on subsequent days. One disadvantage of spacing the drug is that synergistic effects might be missed. On the other hand, a recent study which examined the effect of a simultaneous nitazoxanide-albendazole combination against adult *T. muris in vitro* detected an antagonistic effect [32].

Only a few children were (co)-infected with hookworm and/or *A. lumbricoides*. Nonetheless, these data illustrate that the standard treatment albendazole has a high efficacy against these parasites. The good activity against these two helminths and low efficacy against *T. trichiura* is also supported by the baseline prevalences (*T. trichiura* (82.2%), hookworm (6.8%), and *A. lumbricoides* (4.6%)) observed in the current trial. The settings where the study was conducted (Wawi and Al-Sadik schools) are part of the annual deworming in Zanzibar and are therefore regularly treated with albendazole.

The placebo-controlled treatment group had a cure rate of 55.6% against hookworms. This finding might indicate that the diagnostic tool used to detect hookworm eggs in this study was not sufficiently sensitive. One reason might be that subjects with light infections may only shed very few or sometimes no eggs, resulting in a negative Kato-Katz test result [33], [34]. To overcome this problem in future studies it might be advisable to use an additional diagnostic technique with higher sensitivity such as the FLOTAC technique, which allows using a larger amount of stool, or indirect diagnostic technique such as multiplex real-time PCR [33], [35]–[38].

We observed a high frequency of adverse events, but these were mostly mild, were, at times, already reported before treatment, and also by placebo recipients [39]; and hence were not consistently treatment related. Nevertheless, 3 and 24 hours after the first day of treatment (administration of nitazoxanide or a nitazoxanide-matching placebo) children treated with nitazoxanide reported significantly more adverse events compared to those who had received placebo. This increase in adverse events was not observed after the second day of treatment (children treated with albendazole suffered from a similar number of adverse events episodes than children who had obtained placebo), suggesting that the standard treatment albendazole triggers less adverse events than nitazoxanide. Adverse events related to treatment were resolved 24 hours after the second treatment, and the highest number of adverse events was reported from placebo-treated children.

In conclusion, a single oral dose of nitazoxanide cannot be recommended for the treatment of infection with *T. trichiura* since we observed low cure rate and ERR as well as significantly more adverse events than the standard drug albendazole. Note that, nitazoxanide is also much more expensive than the benzimidazoles, pyrantel pamoate, or levamisole. Moreover, also albendazole showed a low efficacy against *T. trichiura* in our study setting which did also not significantly improve by adding nitazoxanide on the next treatment day. Therefore the discovery and development of novel anthelmintic drugs, in particular against infections with *T. trichiura*, has a high priority.

## Supporting Information

Checklist S1 CONSORT Checklist.
(PDF)

Protocol S1 Trial Protocol.
(DOC)

Table S1 Effect of albendazole, nitazoxanide, sequentially administered albendazole-nitazoxanide combination, and placebo against soil-transmitted helminths calculated with available-case analysis.
(DOC)

Table S2 Number of specific adverse events assessed at different time points.
(DOC)
