# Supplementary material for: Efficacy and Safety of Nitazoxanide, Albendazole, and Nitazoxanide-Albendazole against Trichuris trichiura Infection: A Randomized Controlled Trial
Source: PLoS Negl Trop Dis. 2012 Jun 5;6(6):e1685. doi: 10.1371/journal.pntd.0001685 (PMC3367984; doi:10.1371/journal.pntd.0001685)
Supplement: Protocol S1 — Trial Protocol. (DOC) [file pntd.0001685.s002.doc]

Date: 14.12.2010

Protocol Number: 3-2010

**A randomized double blind trial to assess the efficacy and safety of albendazole, nitazoxanide and albendazole-nitazoxanide in the treatment of *Trichuris trichiura* and other soil transmitted helminth infections in Pemba**

**Investigators**

**Prof. Jennifer Keiser (Pharmacologist; PI)**

Department of Medical Parasitology and Infection Biology

Swiss Tropical and Public Health Institute

Socinstr. 57

CH- 4002 Basel, Switzerland

University of Basel

Petersplatz 1

CH- 4003 Basel, Switzerland

Tel.: +41 61 284-8218

Fax: +41 61 284-8105

E-mail: [jennifer.keiser@unibas.ch](mailto:jennifer.keiser@unibas.ch)

**Prof. Marco Albonico (Parasitologist, Physician, Co-Investigator)**

Ivo de Carneri Foundation

Via IV Marzo 14, 10122 Torino, Italy

University of Torino

Tel +390114310218

Fax +390114361474

Email: albonico@tin.it

**Mr. Said Ali (Co-investigator)**

Director Public Health Laboratory Ivo de Carneri

P.O.BOX 122 Wawi, Chake Chake,

Pemba, Zanzibar(Tanzania)

Tel/Fax +255 24 2452003

Mobile: +255 77 7416867 / 71 2740899

Email: info@phlidc.net

saidmali2003@yahoo.com

**Mr. Shaali Ame (Co-investigator)**

Head Laboratories, Public health Laboratory Ivo de Carneri

P.O.BOX 122 Wawi, Chake Chake,

Pemba, Zanzibar(Tanzania)

Tel/Fax +255 24 2452003

Email: info@phlidc.net

shaaliame@yahoo.com

**Advisors:**

**Prof. Christoph Hatz (Physician, medical expert)**

Medical Department

Swiss Tropical and Public Health Institute

Socinstr. 57

CH- 4002 Basel, Switzerland

Tel.: +41 61 284-8255

Fax: +41 61 284-8283

E-mail: [christoph.hatz@unibas.ch](mailto:christoph.hatz@unibas.ch)

**Prof. Jürg Utzinger (Epidemiologist)**

Department of Public Health and Epidemiology

Swiss Tropical and Public Health Institute

Socinstrasse 57

CH-4002 Basel, Switzerland

Tel: +41 61 284-8129

Fax: +41 61 284-8105

E-mail: [juerg.utzinger@unibas.ch](mailto:juerg.utzinger@unibas.ch)

**Dr. Jan Hattendorf (Statistician, Epidemiologist)**

Department of Public Health and Epidemiology

Swiss Tropical and Public Health Institute

Socinstrasse 57

CH-4002 Basel, Switzerland

Tel:

Fax: +41 61 284-8105

E-mail: [jan.hattendorf@unibas.ch](mailto:jan.hattendorf@unibas.ch)

Sponsor and monitor contact:

Swiss Tropical and Public Health Institute, Socinstr. 57, CH-4002 Basel, Switzerland

**1. Introduction**

The most recent estimates suggest that between 600 and 800 million people are infected with one or several of the common soil-transmitted helminths (STH), *Ascaris lumbricoides*, *T. trichiura,* and hookworms respectively (Hotez et al, 2009). In recent years, the global community has begun to recognize the significance of STH, indeed their global burden might be as high as 39.0 million disability adjusted life years (DALYs) lost each year, with hookworm infection alone causing the loss of 22.1 million DALYs. The symptoms of STH infections are nonspecific and may only be apparent in cases of heavy infection. Infection is typically most intense and debilitating in school-aged children. Chronically infected children suffer from malnutrition, physical and intellectual growth retardation, and cognitive and educational deficits (Bethony et al., 2006).

At present, there are four drugs on the World Health Organization (WHO) model list of essential medicines and they have been widely and effectively used against STH infections for three decades or more: two benzimidazoles (albendazole and mebendazole), levamisole and pyrantel pamoate (WHO, 2002, 2009; Utzinger and Keiser, 2004; Keiser and Utzinger, 2008). Today, the two benzimidazoles are the most commonly used drugs for the treatment and control of soil-transmitted helminthiases, and they are increasingly utilized in preventive chemotherapy, which is the regular administration of anthelminthic drugs to at-risk populations (e.g. school-aged children) usually without prior diagnosis. Preventive chemotherapy is advocated by WHO and other organizations because of its rapid impact on morbidity, and the fact that the drugs that can be administered by personnel outside the health sector (e.g. teacher) independent of recipients body weight (e.g. albendazole is administered at a single oral dose of 400 mg) (WHO, 2006).

Repeated use of only a few drugs over a long period of time raises a concern that resistance to these drugs may develop and spread. Worryingly, recent reports have indicated unexpectedly low cure rates using mebendazole against hookworm infection in different epidemiological settings, first in Mali, then in Zanzibar, and most recently in Vietnam (Flohr et al. 2007). Although further research is needed to confirm whether these results indicate the development of anthelminthic drug resistance, however, experience from the veterinary practice raises a caution that drug resistance may appear quickly under drug pressure and become irreversibly dominant in helminth populations (Keiser and Utzinger, 2010).

In addition, in terms of cure rate, treatment of *T. trichiura* with single oral doses of current anthelminthics is not satisfactory; observed cure rates ranged between 10% and 36%. Egg reduction rates ranged from 0 to 93% (Keiser and Utzinger, 2008).

The lack of concerted efforts in anthelminthic drug development for human use, combined with the growing concerns on emerging drug resistance in the face of imminent large-scale deployment of benzimidazoles and low cure rates observed in the treatment of *T. trichiura*, implicit an urgent need to develop safe and effective new anthelmintic drugs or a drug combination against human STH to complement the existing armament. Mathematical modeling revealed that the likelihood of anthelminthic resistance development is significantly delayed when drug mixtures are administered (Barnes et al., 1995). Drug combinations are widely used in the treatment of malaria, tuberculosis or HIV (Zhang, 2007). Interestingly though, only few human clinical trials have tested combinations of approved anthelminthic drugs to explore whether they exhibit additive, synergistic or antagonistic effects.

Nitazoxanide is an antiprotozoal drug used for the treatment of infections with *Cryptosporidium parvum* and *Giardia intestinalis*. The drug is commonly given in six divided doses (500 mg bid for 3 days for adults and 200 mg bid for 3 days for children aged 4-11 years). The safety and tolerability of nitazoxanide in humans has been documented by > 10 years of commercial use. More than 20 million people have been treated with nitazoxanide in post-marketing experience (Romark Laboratories, pers. commun.), most of them for relatively short durations ranging from 3 to 10 days. Three studies carried out in Mexico have shown that nitazoxanide achieved high cure rates against *T. trichiura* and *A. lumbricoides* (Romero Cabello et al., 1997; Juan et al., 2002; Diaz et al., 2003). Nitazoxanide, however, shows only moderate efficacy against hookworms (Romark Laboratories, unpublished observation). While two of the above mentioned studies testing nitazoxanide against STH used a 3-day dose regimen, the treatment schedule was not described in one of the studies. The efficacy of single dose treatment of nitazoxanide against STH has not been described. However, a study comparing a single dose of nitazoxanide (1.2 g) versus multiple doses (15 mg/kg/day for 3 days) found that the single dose was highly efficacious against intestinal parasites and better tolerated (Belkind-Valdovinos et al., 2004). Since drugs for treating STH infections are mostly administered in the framework of preventive chemotherapy programs it is important to administer these drugs using single doses.

**2. Hypothesis and study aim**

Hypothesis: An albendazole- nitazoxanide combination achieves a higher efficacy against *T. trichiura* than single albendazole.

Study aim: To comparatively assess the efficacy and safety of oral albendazole (single 400-mg dose), oral nitazoxanide (single 1000-mg dose) and a nitazoxanide-albendazole combination (1000 mg - 400 mg) against *T. trichiura* and other STH and intestinal protozoan infection among 500 school-aged children in Pemba in a randomized double blind four arm trial. One group of children will be administered placebo. In the present study the combination of nitazoxanide and albendazole will be administered on subsequent days (respecting the half life of the drugs—nitazoxanide has a half life of 7 hours and albendazole a half life of 8-12 h) since drug interactions have not been studied. This study can provide the basis for potential future studies using fixed dose combinations.

**3. Experimental design**

*3.1. Study area and population*

The study will be carried out in school-aged children (6-10 years old) in Pemba, United Republic of Tanzania in an area with a high *T. trichiura* prevalence.

*3.2 Participant inclusion criteria*

1. Written informed consent signed by parents and/or legal guardian.
2. Able and willing to be examined by a study physician at the beginning and at the end of the study (3 weeks post-treatment)
3. Able and willing to provide 2 stool samples at the beginning and at the end of the study.
4. Absence of major systemic illnesses (e.g. cancer, diabetes, clinical malaria or hepato-splenic schistosomiasis) as assessed by the medical doctor, upon initial clinical assessment.
5. No known or reported history of chronical illness as cancer, diabetes, chronic heart, liver or renal disease.
6. No recent anthelminthic treatment (within past 4 weeks).

*3.3. Participant exclusion criteria*

1. No written informed consent by parents/legal guardian and child.
2. Presence of any abnormal medical condition, judged by the study physician.
3. History of acute or severe chronic disease.(cancer, diabetes, chronic heart, liver or renal disease)
4. Recent use of anthelminthic drug (within past 4 weeks).
5. Attending other clinical trials during the study.

Participants who were diagnosed with a STH infection, but who were excluded from the study due to one or several of the above mentioned exclusion criteria will be offered a single oral dose of 400 mg albendazole.

*3.4 Criteria for discontinuation*

A subject can be discontinued from the study for the following reasons:

1. Withdraws voluntarily from the study.

2. At the discretion of the Principal Investigator if the participant is not compliant to the requirements of the protocol.

*3.5 Baseline survey*

The medical history of STH-infected school-aged children participating in the study will be assessed with a standardized and previously used questionnaire, in addition to a full clinical examination carried out by the study clinician.

Two stool samples will be collected on subsequent days. The Kato-Katz technique will be used for the quantitative assessment of *T. trichiura* and other STH infections (Katz et al., 1972). Two Kato-Katz thick smears (41.7 mg) will be prepared from each stool sample. The slides will be analyzed by experienced technicians and a subsequent independent quality control of sample results will be conducted. Infection intensity (expressed as the arithmetic mean egg count per gram of stool (EPG) will be calculated for each individual. In addition, 1–2 g of stool will be transferred to a 15 ml Falcon plastic tube and conserved in 10 ml of a sodium acetate–acetic acid-formalin (SAF) for subsequent ether-concentration and FLOTAC examination (Knopp et al., 2009, Cringoli et al, 2010, Utzinger et al., 2010) to determine intestinal nematode and protozoan infections. In brief, each sample will be split into two aliquots, one subjected to the ether-concentration method using standard protocols (Utzinger et al., 2010) and the second subjected to the FLOTAC-400 dual technique (Cringoli et al., 2010). Finally, a stool sample (0.5 g) will be transferred into an Eppendorf tube and frozen (-20 C) for subsequent PCR analysis for STH.

*3.6 Treatment and randomization*

Nitazoxanide (Alinia®) 500 mg tablets will be either obtained from Romark Laboratories or purchased from the international pharmacy (Zurich, Switzerland) and albendazole 400 mg tablets will be purchased from Inresa (Bartenheim, France). Placebo tablets will be purchased or produced by the Department of Pharmaceutical Sciences, University of Basel. Placebo tablets will look identical to albendazole and nitazoxanide tablets.

The drug administration is depicted in Figure 1.

Group 1 will receive two tablets of nitazoxanide (500 mg each) on the first day and one albendazole matching placebo on the next day. Group 2 will receive two tablets of nitazoxanide matching placebo on the first day and one tablet of albendazole (400 mg) on the next day. Group 3 will receive two tablets of nitazoxanide (500 mg each) on day 1 and one albendazole tablet on day 2. Group 4 will be administered 2 tablets of nitazoxanide matching placebo on the first day and 1 albendazole placebo tablet on the next day. Since we administer the drugs on subsequent days drug interactions are not expected. The half life of albendazole is 8-12 hours (package insert Zentel®). The terminal half life of nitazoxanide is 7 hours. In vitro studies have shown that tizoxanide (the main metabolite of nitazoxanide) has no significant effect on on cytochrome P450 enzymes, hence drug interactions are unlikely (package insert Alinia®).

Study participants will be randomly assigned to one of the four treatment arms using a computer generated randomization code. The codes will be held on a central server. The blinding will be maintained throughout the trial until data entry and processing are complete and the data has been verified. Following release of the final data the randomization codes will be released. The study randomization code will only be broken for valid medical or safety reasons.

All drugs will be administered in the presence of the study physician, and ingestion confirmed. This will be recorded with the time and date of closing. Subjects will be asked not to take any drugs other than those prescribed by the study medical team. After ingestion of the medication, the subjects will be observed for 1 hour to ensure retention of the drug. Vomiting within 1-hour post-dosing will require re-dosing. The subjects will not allow more than one repeated dose. No re-administration will be needed for subjects vomiting after 1 hour.

The principal investigator is responsible for drug accountability at the study site. Maintaining drug accountability includes careful and systematic study drug storage, handling, dispensing, and documentation of administration.

*Figure 1: Drug administration*

*Figure 2: Study flow*

*3.7. Sample size and statistical analysis*

Sample size:

Testing for an interaction in a 2 x 2 factorial design requires a greatly increased sample size. Since interaction is not our primary interest, we ensured to have adequate power to detect main effects in the presence of an interaction, i.e. to analyze the trial as a four-arm parallel-group design.

We assume that the cure rate of albendazole against *T. trichiura* infections is 28% based on a recent systematic literature review and meta-analysis (Keiser et al. 2008). The efficacy of a single dose of nitazoxanide has not been assessed so far; we assume that it is in the same order (cure rates of 20-30%). Moreover, we hypothesize that an albendazole-nitazoxanide combination achieves a cure rate of 50%. Assuming an imperfect test with a sensitivity of 90%, Monte Carlo simulations revealed that we require 95 infected individuals in each group to detect a difference of single medication treatments versus both, the placebo group and the albendazole-nitazoxanide group at a significance level of 5% with 80% power. The sample size was further increased by 30 children per group to account for inclusion of *T. trichiura* negative children (we estimate a *T. trichiura* prevalence of 80-90%) and potential losses to follow-up resulting in a total of 500 study participants (125 per group).

Data analysis:

Both intention to treat and per protocol analyses will be conducted. Logistic regression models will be used to examine cure rates among different treatment arms. Negative binomial regression will be applied to calculate the incidence rate ratio (IRR) between the numbers eggs recovered in stool examination among group treatment. Pearson’s x2 test will be applied to compare baseline binary characteristics and the proportion of the reported adverse events between treatment arms. Statistical significance will be estimated using a likelihood ratio test. Negative binominal models will be fitted to compare the number of adverse event among the treatment groups. P-value below 5% will be considered significant.

*3.8 Adverse events*

Definitions:

*Adverse event*: any untoward medical occurrence, regardless of the causal relationship of the event with the study treatments.

*Associated with the use of the study treatments*: there is a reasonable possibility that the adverse event may have been caused by the study treatment.

*Disability*: a substantial disruption of a person’s ability to conduct normal life functions.

*Life-threatening adverse event*: any adverse event that places the participant or subject, in the view of the investigator, at immediate risk of death.

*Serious adverse event*: any adverse event occurring at any dose that results in any of the following outcomes: death, a life-threatening adverse event, in participant hospitalization or prolongation of existing hospitalization, a persistent or significant disability/incapacity, or a congenital anomaly/birth defect. Hospitalization shall include any initial admission (even if less than 24 hours) to a healthcare facility as a result of a precipitating clinical adverse event; to include transfer within the hospital to an intensive care unit. Hospitalization or prolongation of hospitalization in the absence of a precipitating, clinical adverse event (e.g., for a preexisting condition not associated with a new adverse event or with a worsening of the preexisting condition; admission for a protocol-specified procedure) is not, in itself, a serious adverse event.

*Unexpected adverse event*: Any adverse event, the frequency, specificity or severity of which is not consistent with the risk information described for these drugs.

Recording adverse events following albendazole and nitazoxanide:

Few adverse events have been reported following nitazoxanide administration. The most common adverse events were abdominal pain, diarrhea, nausea and headache (Anderson et al. 2007). Similar adverse events have also been reported for albendazole, which are self-limiting and do not interfere with daily activities (Keiser and Utzinger, 2008). Participants receiving albendazole, nitazoxanide and an albendazole-nitazoxanide combination will be visited daily in the mornings at the school by the study physician during the period of drug administration (during 2 days). Children will be kept for observation for at least 3 hours following treatment for any acute adverse events. If there is any abnormal finding, the study physicians will perform a full clinical examination on each individual and findings will be recorded. An emergency kit will be available on site to treat any urgent side effects. Children will also be interviewed on the following day (24 h post-treatment) for the occurrence of adverse events.

Information on all adverse events (incidence, onset, cessation, duration, intensity, frequency, seriousness and causality) will be entered immediately in the source document, and also in the appropriate adverse event module of the case report form (CRF). For all adverse events, sufficient information will be pursued and/or obtained so as to permit 1) an adequate determination of the outcome of the event (i.e., whether the event should be classified as a *serious adverse event*) and; 2) an assessment of the casual relationship between the adverse event and the study treatments. Intensity of adverse events will be judged by the study physician as followed:

*Mild:* present but no action required.

*Moderate:* (i) present and require medication for symptomatic relief as requested by the affected people or (ii) present and interfering with normal daily activities.

*Severe:* present and require medical intervention beyond symptomatic relief.

*Serious* (see definition above)

The clinical course of each event will be followed until resolution, stabilization, or until it has been determined that the study treatment or participation is not the cause. Serious adverse events that are still ongoing at the end of the study period will be followed up to determine the final outcome. Any serious adverse event that occurs after the study period and is considered to be possibly related to the study treatment or study participation will be recorded and reported immediately. Children experiencing serious adverse events on the first treatment day will be excluded from the study.

Reporting of serious adverse events:

Any study-related unanticipated problem posing risk of harm to subjects or others, and any type of serious adverse event, will be reported to the study sponsor by telephone within 24 hours of the event. To report such events, a Serious Adverse Event (SAE) form must be completed by the investigator and faxed to the study sponsor within 24 hours. The investigator will keep a copy of this SAE form on file at the study site. Report serious adverse events by phone and facsimile to the Zanzibar Ministry of Health and Social Welfare:

Prof. Jennifer Keiser

Department of Medical Parasitology and Infection Biology

Swiss Tropical and Public Health Institute

Socinstr. 57

CH- 4002 Basel, Switzerland

University of Basel

Petersplatz 1

CH- 4003 Basel, Switzerland

Tel.: +41 61 284-8218

Fax: +41 61 284-8105

E-mail: jennifer.keiser@unibas.ch

Within the following 48 hours, the investigator must provide further information on the serious adverse event or the unanticipated problem in the form of a written narrative. This should include a copy of the completed Serious Adverse Event form, and any other diagnostic information that will assist the understanding of the event. Significant new information on ongoing serious adverse events should be provided promptly to the study sponsor.

*3.9 Endpoint parasitological survey and clinical evaluation*

Primary endpoint: Cure and egg reduction rate

Secondary endpoint: Safety and tolerability

Starting at 21 days after the last treatment dose has been administered, participants stool samples will again be examined for the presence of *T. trichiura* and other STH eggs and intestinal protozoan in 2 different stool samples (collected over a maximum of 3 days) using the same diagnostic approach as during the baseline survey. Children will be considered *T. trichiura* and STH-negative if no eggs have been found in 2 stool specimens analyzed by duplicate Kato-Katz thick smears per specimen. Examination of SAF-preserved stool samples with the ether concentration and the FLOTAC techniques for intestinal protozoan will be utilized to assess the effect of treatment on intestinal protozoa.

At the end of the study (24 days post-treatment), all children diagnosed with soil-transmitted helminths will receive a single 400 mg oral dose of albendazole and participants diagnosed with intestinal protozoan will be treated with multiple doses of nitazoxanide.

*3.10 Concomitant therapy*

All medications taken one month before and during the study period must be recorded with indication, dose regimen, date and time of administration.

### Medication(s)/treatment(s) permitted during the trial

- Analgesics and antipyretics are allowed to be given to the subjects in case of fever, antiemetics to prevent nausea and vomiting and/or antibiotics to prevent or treat bacterial superinfection.

### Medication(s)/treatment(s) NOT permitted during the trial

- No other active drugs against helminths are permitted during the trial

**4. Data Quality Assurance**

The investigators are responsible for an adequate data quality. Prior to the initiation of the study, an short investigator’s meeting will be held with the investigators and their

study coordinators and a member from Swiss TPH. This meeting will include a detailed discussion of the protocol, performance of study procedures, CRF completion, and specimen collection and diagnostic methods. In addition to the investigator’s meeting, the study personnel at each site will be trained on the study procedures. We will include a clinical monitor in our study (Dr. Rahila). An independent quality control of sample results will be conducted for the Kato-Katz and Flotac analysis. In addition, a review of the data will be conducted. All data hand entered in the database will be verified by a double-key entry procedure. Any discrepancies will be reviewed against the hard copy CRF and corrected.

**5. Ethical considerations**

The two drugs which are compared and combined are well known, widely used and have little adverse events (WHO, 2002, Rossignol et al., 2006). All children enrolled in the study will benefit from a treatment. All diagnosed parasitic infections will be treated according to the national guidelines.

The study will be submitted for approval by the Institutional Research commission of the Swiss Tropical and Public Health Institute (Swiss TPH) and the Medicines Research Department at the Swiss TPH (Basel, Switzerland) and the Public health Laboratory -Ivo de Carneri in Pemba. Ethical clearance will be sought from the Ethics Committee of Basel (EKBB, Basel, Switzerland) and the Ministry of Health and Social Welfare (MoHSW, Zanzibar). The study will be undertaken in accordance with the Declaration of Helsinki and good clinical practice (GCP). Parents or legal guardians of eligible children will be asked to sign a written informed consent sheet. Community meetings will be conducted to explain the purpose of the study, procedures and to obtain written informed consent. Participation is voluntary and children have the right to withdraw from the study at any given point in time with no further obligations.

Confidentiality of information will be assured to the participants. The investigators have all been trained in GCPs (copy of proofs attached).

**6. Compliance**

The investigator team is responsible for ensuring that the protocol is strictly followed. The investigator should not make any changes without the agreement of the PI and CoPI, except when necessary to eliminate an apparent immediate hazard or danger to a subject. The investigator will work according to the protocol and GCP. The investigator may take any steps judged necessary to protect the safety of the participants, whether specified in the protocol or not. Any such steps must be documented. During the treatment the records are maintained by the responsible medical doctor. All entries have to be made clearly readable with a pen. The investigator must be thoroughly familiar with the properties, effects and safety of the investigational pharmaceutical product.

# . Data handling

Information about study subjects will be kept confidential and managed accordingly. A Case Report Form (CRF) will be completed for each subject enrolled into the clinical study. The investigators will review, approve and sign/date each completed CRF; the investigator-sponsor’s signature serving as attestation of the investigator-sponsor’s responsibility for ensuring that all clinical and laboratory data entered on the CRF are complete, accurate and authentic. The study case report form (CRF) is the primary data collection instrument for the study. All data requested on the CRF must be recorded. All missing data must be explained. If a space on the CRF is left blank because the procedure was not done or the question was not asked “N/D” will be entered. If the item is not applicable to the individual case “N/A” will be written. All entries will be printed in black ink. All corrections must be initialed and dated. *Source Data* are the clinical findings and observations, laboratory data maintained at the study site. Source data are contained in source documents. *Source Documents* are the physician’s subject records maintained at the study site. When applicable, information recorded on the CRF shall match the *Source Data* recorded on the *Source Documents*. Essential documents will be kept for at least 2 years.

**8. Publication Policy**

The final results of this study will be published in a scientific journal and presented at scientific conferences. All results from this investigation are considered confidential and shall not be made available to any third part by any member of the investigating team before publication.

**9. Financing and insurance**

The study will be financed through the Swiss national Science Foundation professorship grant of Jennifer Keiser. A general liability insurance of the Swiss TPH is in place (Winterthur Police Nr. 4746321)

**10. References**

Anderson, VR; Curran, MP. Nitazoxanide: A review of its use in the treatment of gastrointestinal infections. Drugs. 2007; **67**: 1947-1967.

Barnes EH, Dobson RJ, Barger IA. Worm control and anthelmintic resistance: adventures with a model. *Parasitol Today*. 1995; **11**: 56-63.

Belkind-Valdovinos U, Belkind-Gerson J, Sanchez-Francia D *et al.* Nitazoxanide vs albendazole against intestinal parasites in a single dose and for three days. *Salud Publica De Mexico*. 2004; **46**: 333-40.

Bethony J, Brooker S, Albonico M et al. Soil-transmitted helminth infections: ascariasis, trichuriasis, and hookworms. *Lancet*. 2006; **367**: 1521-32.

Cringoli G, Rinaldi L, Maurelli MP *et al.* FLOTAC: new multivalent techniques for qualitative and quantitative copromicroscopic diagnosis of parasites in animals and humans. *Nat Protoc*. 2010; **5**: 503-15.

Diaz E, Mondragon J, Ramirez E et al. Epidemiology and control of intestinal parasites with nitazoxanide in children in Mexico. *Am J Trop Med Hyg*. **2003**; 68: 384-5.

Flohr, C., Tuyen, L.N., Lewis, S., Minh, T.T., Campbell, J., Britton, J., Williams, H., Hien, T.T., Farrar, J., and Quinnell, R.J. Low efficacy of mebendazole against hookworms in Vietnam: Two randomized controlled trials. *Am J Trop Med Hyg*. 2007; **76**: 732-736.

Juan JO, Lopez Chegne N, Gargala G *et al.* Comparative clinical studies of nitazoxanide, albendazole and praziquantel in the treatment of ascariasis, trichuriasis and hymenolepiasis in children from Peru. *Trans R Soc Trop Med Hyg*. 2002; **96**: 193-6.

Hotez PJ, Fenwick A, Savioli L et al. Rescuing the bottom billion through control of neglected tropical diseases. *Lancet*. 2009; **373**: 1570-5.

Keiser J, Utzinger J. Efficacy of current drugs against soil-transmitted helminth infections: systematic review and meta-analysis. *JAMA*. 2008; **299**: 1937-48.

Katz N, Chaves A, Pellegrino J. A simple device for quantitative stool thick-smear technique in schistosomiasis mansoni. *Rev. Inst. Med. Trop. Sao Paulo*. 1972; **14**: 397-400.

Keiser J, Utzinger J. The drugs we have and the drugs we need against major soil-transmitted helminths infection. *Adv Parasitol*. 2010; **73**:197-230.

Knopp S, Glinz D, Rinaldi L *et al.* FLOTAC: a promising technique for detecting helminth eggs in human faeces. *Trans R Soc Trop Med Hyg*. 2009; **103**: 1190-4.

Romero Cabello R, Guerrero LR, Munoz Garcia MR et al. Nitazoxanide for the treatment of intestinal protozoan and helminthic infections in Mexico. *Trans R Soc Trop Med Hyg*. 1997; **91**: 701-3.

Rossignol JF, Abu-Zekry M, Hussein A et al. Effect of nitazoxanide for treatment of severe rotavirus diarrhoea: randomised double-blind placebo-controlled trial. *Lancet*. 2006; **368**: 124-9.

Utzinger J, Keiser J. Schistosomiasis and soil-transmitted helminthiasis: common drugs for treatment and control. *Exp Opin Pharmacother*. 2004; **5**: 263-85.

Utzinger J, Botero-Kleiven S, Castelli F et al. Microscopic diagnosis of SAF-fixed stool samples for helminths and intestinal protozoa: a comparison among European reference laboratories. Clin. Microbiol. Infect. 2010; **16**: 267-73.

WHO, *Prevention and control of schistosomiasis and soil-transmitted helminthiasis: report of a WHO expert committee*. WHO Tech Rep Ser 912. Vol. No. 912. 2002, Geneva: World Health Organization. 1-57.

WHO, *Preventive chemotherapy in human helminthiasis: coordinated use of anthelminthic drugs in control interventions: a manual for health professionals and programme managers*. 2006, World Health Organization: Geneva.

WHO, WHO Model formulary 2008, M.C. Stuart, M. Kouimtzi, and R.S. Hill, Editors. 2009: World Health Organization Geneva.

Zhang, Y.;Advances in the treatment of tuberculosis, Clin Pharmacol Ther. 2007; **82**: 595-600.
