# Supplementary material for: Efficacy and Safety of Nitazoxanide, Albendazole, and Nitazoxanide-Albendazole against Trichuris trichiura Infection: A Randomized Controlled Trial
Source: PLoS Negl Trop Dis. 2012 Jun 5;6(6):e1685. doi: 10.1371/journal.pntd.0001685 (PMC3367984; doi:10.1371/journal.pntd.0001685)
Supplement: Table S1 — Effect of albendazole, nitazoxanide, sequentially administered albendazole-nitazoxanide combination, and placebo against soil-transmitted helminths calculated with available-case analysis. (DOC) [file pntd.0001685.s003.doc]

**Supplementary Table 1: Effect of albendazole, nitazoxanide, sequentially administered albendazole-nitazoxanide combination, and placebo against soil-transmitted helminths calculated with available-case analysis.**

| **Characteristic** | **Nitazoxanide + albendazole** | **Only albendazole** | **Only nitazoxanide** | **Only placebo** |
| --- | --- | --- | --- | --- |
| ***Trichuris trichiura*** |  |  |  |  |
| No. of persons positive before treatment | 134 | 135 | 139 | 139 |
| No. of persons not cured after treatment | 113 | 116 | 130 | 127 |
| Cure rate % (95% CI) | 15.7 | 14.1 | 6.5 | 8.6 |
|  | (9.4–21.9) | (8.1–20.0) | (2.3–10.6) | (3.9–13.4) |
| **Hookworm** |  |  |  |  |
| No. of persons positive before treatment | 14 | 11 | 13 | 9 |
| No. of persons not cured after treatment | 2 | 2 | 4 | 4 |
| Cure rate % (95% CI) | 85.7 | 81.8 | 69.2 | 55.6 |
|  | (64.7–100.0) | (54.6–100.0) | (40.2–98.3) | (15.0–96.1) |
| ***Ascaris lumbricoides*** |  |  |  |  |
| No. of persons positive before treatment | 5 | 9 | 8 | 7 |
| No. of persons not cured after treatment | 0 | 0 | 3 | 7 |
| Cure rate % (95% CI) | 100.0 | 100.0 | 62.5 | 0.0 |
|  |  |  | (19.2–100.0) |  |

Children who missed a treatment but had outcome data were included.
