# Supplementary material for: Efficacy and Safety of Nitazoxanide, Albendazole, and Nitazoxanide-Albendazole against Trichuris trichiura Infection: A Randomized Controlled Trial
Source: PLoS Negl Trop Dis. 2012 Jun 5;6(6):e1685. doi: 10.1371/journal.pntd.0001685 (PMC3367984; doi:10.1371/journal.pntd.0001685)
Supplement: Table S2 — Number of specific adverse events assessed at different time points. (DOC) [file pntd.0001685.s004.doc]

**Supplementary Table 2: Number of specific adverse events, stratified by treatment arm, as assessed at different time points**

|  | **Before treatment** | | | | | **3 hours after first treatment** | | | | | **24 hours after first treatment** | | | | | **3 hours after second treatment** | | | | | **24 hours after second treatment** | | | | | **Total (%)^b^** |
| --- | --- | --- | --- | --- | --- | --- | --- | --- | --- | --- | --- | --- | --- | --- | --- | --- | --- | --- | --- | --- | --- | --- | --- | --- | --- | --- |
|  |  | **Treatment arm** | | | |  | **Treatment arm** | | | |  | **Treatment arm** | | | |  | **Treatment arm** | | | |  | **Treatment arm** | | | |  |
| **Adverse event** | Overall | 1 | 2 | 3 | 4 | Overall | 1 | 2 | 3 | 4 | Overall | 1 | 2 | 3 | 4 | Overall | 1 | 2 | 3 | 4 | Overall | 1 | 2 | 3 | 4 |  |
| Abdominal cramps | 13 | 3 | 1 | 5 | 4 | 68 | 20 | 11 | 24 | 13 | 32 | 14 | 4 | 10 | 4 | 34 | 8 | 8 | 14 | 4 | 31 | 9 | 7 | 6 | 9 | 165 (53.7) |
| Headache | 10 | 5 | 2 | 1 | 2 | 26 | 10 | 6 | 4 | 6 | 17 | 5 ^a^ | 3 | 6 | 3 | 14 | 4 | 2 | 3 | 5 | 12 | 2 | 3 | 6 | 1 | 69 (22.5) |
| Nausea | 6 | 1 | 2 | 2 | 1 | 5 | 2 | 0 | 2 | 1 | 5 | 2 | 0 | 2 | 1 | 8 | 2 | 2 | 3 | 1 | 3 | 1 | 0 | 0 | 2 | 21 (6.8) |
| Vertigo | 3 | 1 | 1 | 0 | 1 | 9 | 2 | 2 | 5 | 0 | 4 | 1 | 0 | 3 | 0 | 2 | 0 | 0 | 0 | 2 | 2 | 0 | 1 | 1 | 0 | 17 (5.5) |
| Diarrhea | 2 | 0 | 2 | 0 | 0 | 1 | 0 | 0 | 1 | 0 | 7 | 2 | 1 | 4 | 0 | 1 | 1 | 0 | 0 | 0 | 5 | 0 | 3 | 0 | 2 | 14 (4.6) |
| Fever | 0 | 0 | 0 | 0 | 0 | 3 | 3 | 0 | 0 | 0 | 2 | 1 | 0 | 1 | 0 | 2 | 2 | 0 | 0 | 0 | 4 | 2 | 0 | 1 | 1 | 11 (3.6) |
| Allergic reaction | 2 | 1 | 1 | 0 | 0 | 1 | 0 | 0 | 0 | 1 | 2 | 0 | 1 | 1 | 0 | 0 | 0 | 0 | 0 | 0 | 2 | 0 | 1 | 0 | 1 | 5 (1.6) |
| Vomiting | 0 | 0 | 0 | 0 | 0 | 2 | 1 | 0 | 1 | 0 | 0 | 0 | 0 | 0 | 0 | 0 | 0 | 0 | 0 | 0 | 2 | 1 | 1 | 0 | 0 | 4 (1.3) |
| Fatigue | 0 | 0 | 0 | 0 | 0 | 0 | 0 | 0 | 0 | 0 | 1 | 0 | 1 | 0 | 0 | 0 | 0 | 0 | 0 | 0 | 0 | 0 | 0 | 0 | 0 | 1 (0.3) |
|  |  |  |  |  |  |  |  |  |  |  |  |  |  |  |  |  |  |  |  |  |  |  |  |  |  |  |
| Total | 36 | 11 | 9 | 8 | 8 | 115 | 38 | 19 | 37 | 21 | 70 | 25 | 10 | 27 | 8 | 61 | 17 | 12 | 20 | 12 | 61 | 15 | 16 | 14 | 16 | 307 (100) |

^a^ 1 adverse event was classified as moderate

^b^ Total number of adverse events does not include adverse events observed before treatment

Adverse events were assessed at five different time points (before treatment, 3 and 24 hours after first treatment, and 3 and 24 hours after second treatment). Nitazoxanide was given on the first day of treatment, while albendazole was given on the second day of treatment. Treatment arms are the following: 1, nitazoxanide-albendazole combination, administered sequentially over two consecutive days; 2, albendazole alone; 3, nitazoxanide alone; 4, placebo.
